# Real-Time, Transcranial Monitoring of Safe Blood-Brain Barrier Opening in Non-Human Primates

**DOI:** 10.1371/journal.pone.0084310

**Published:** 2014-02-05

**Authors:** Fabrice Marquet, Tobias Teichert, Shih-Ying Wu, Yao-Sheng Tung, Matthew Downs, Shutao Wang, Cherry Chen, Vincent Ferrera, Elisa E. Konofagou

**Affiliations:** 1 Departments of Biomedical Engineering, Columbia University, New York, New York, United States of America; 2 Department of Neuroscience, Columbia University, New York, New York, United States of America; 3 Department of Radiology, Columbia University, New York, New York, United States of America; Institute of Neurology (Edinger-Institute), Germany

## Abstract

The delivery of drugs to specific neural targets faces two fundamental problems: (1) most drugs do not cross the blood-brain barrier, and (2) those that do, spread to the entire brain. To date, there exists only one non-invasive methodology with the potential to solve these problems: selective blood-brain barrier (BBB) opening using micro-bubble enhanced focused ultrasound. We have recently developed a single-element 500-kHz spherical transducer ultrasound setup for targeted BBB opening in the non-human primate that does not require simultaneous MRI monitoring. So far, however, the targeting accuracy that can be achieved with this system has not been quantified systematically. In this paper, the accuracy of this system was tested by targeting caudate nucleus and putamen of the basal ganglia in two macaque monkeys. The average lateral targeting error of the system was ∼2.5 mm while the axial targeting error, i.e., along the ultrasound path, was ∼1.5 mm. We have also developed a real-time treatment monitoring technique based on cavitation spectral analysis. This technique also allowed for delineation of a safe and reliable acoustic parameter window for BBB opening. In summary, the targeting accuracy of the system was deemed to be suitable to reliably open the BBB in specific sub-structures of the basal ganglia even in the absence of MRI-based verification of opening volume and position. This establishes the method and the system as a potentially highly useful tool for brain drug delivery.

## Introduction

To date, there exists no non-invasive clinical method to deliver drugs to specifically targeted brain regions mainly because of the presence of the Blood-Brain Barrier (BBB) [1]–[2]. There are a number of methods currently available such as intracranial injections, mixing or attaching agents to BBB-modifying chemicals as well as the chemical alteration of agents to be delivered through endogenous transport systems [3]. However, these techniques are either invasive, drug-specific or are plagued by very poor spatial specificity. This is particularly problematic for deep sub-cortical structures such as the basal ganglia because alternative invasive approaches may damage not only the target structure, but also overlying grey and white matter. A non-invasive method would be highly useful not only for clinical use, but also for neuroscientists trying to study the role of the basal ganglia in animal models of neurological and neuropsychiatric disorders such as Parkinson's, Huntington's Schizophrenia, Obsessive Compulsive Disorder and addiction.

Here, we present recent advances in the development of a focused ultrasound (FUS) method for targeted drug delivery in the non-human primate, highlighting the ability of the method to reliably target specific sub-structures of the basal ganglia. We show that the method has a number of desirable features that make it highly interesting not only for clinicians interested in delivering therapeutic drugs to the basal ganglia, but also for neuroscientists trying to manipulate the behavior of certain neuronal populations to study their role in normal function and models of disease.

The method uses brief FUS pulses in combination with systemically injected micro-bubbles to cause reversible increases in the permeability of localized parts of the BBB. The BBB is a selective barrier within the neurovascular unit formed by the endothelial cells that line the cerebral microvessels. The BBB hinders the effective systemic delivery to the brain of more than 98% of small-molecule drugs and nearly all large-molecule drugs [3]. Mixing or attaching agents to BBB-modifying chemicals, and the chemical alteration of agents enables them to be delivered through endogenous transport systems [4] but those techniques are not region specific. It is therefore a risky procedure, as it increases the influx of therapeutic molecules throughout the brain. The FUS-method locally increases the permeability of the BBB by low shear stress caused by micro-bubbles oscillating between the walls of brain capillaries in the focused ultrasound beam [5]. The mechanical stress is believed to enable passive diffusion of compounds that would not cross the intact BBB. This technique has been proven capable of delivering compounds with a wide range of size. Choi et al. [6], proved that 70 kDa Dextran was able to be delivered using FUS and Wang et al. [7] delivered adeno-associated viruses (4 MDa) using the same technique. The ultrasound procedure is completely non-invasive and can be delivered through the intact skin and skull. The focusing of the ultrasound prevents high pressures outside of the focus and hence restricts the opening of the BBB to the ultrasound focus. Therefore, the method is especially well suited to target deep subcortical brain structures while leaving the overlying cortex unaffected.

One of the main challenges in the development of the FUS method for clinical use as well as the laboratory setting is to maximize targeting accuracy while minimizing the time and effort necessary for accurate targeting. Targeting accuracy is limited primarily by the skull that causes aberrations of the ultrasound beam. The discrepancy between the high speed of sound through the skull and the low speed through the underlying brain tissue, combined with a severe attenuation of ultrasound waves through the skull bone, strongly distorts the beam shape especially at higher frequencies [8]. Moreover, the trabecular layer of the skull induces heterogeneities in both speed of sound and density that lead to strong phase aberrations of the acoustic beam. At higher frequencies the defocusing effect of the skull is more severe as the wavelength tends to reach the size of local skull bone heterogeneities (typical size of the trabeculae is around 1 mm). The phase aberrations can be reduced by reducing the ultrasound frequency. However, it also increases the size of the focal region and the likelihood of inertial cavitation that may cause permanent tissue damage [9]. We have previously shown that intermediate ultrasound frequencies of 500-kHz allow safe and reversible BBB opening in non-human primates [10], [11]. The resulting wavelength of 3 mm at this intermediate frequency may provide a good trade-off between targeting accuracy on the one hand and safety on the other, without requiring phase aberration correction [12].

Another important challenge in therapeutic ultrasound remains the need for real-time monitoring and treatment efficiency verification. It has been shown that a passive cavitation detector (PCD) can be used to transcranially acquire the acoustic emissions stemming from the microbubble [13]. The frequency analysis of the backscattered signal and, more specifically the cavitation dose, has been proven relevant to characterizing the bubble-capillary interaction and potential damage in mice along with complete histological analysis [10], [14]. These studies determined acoustic parameters (in situ pressure, pulse length and pulse repetition frequency) as well as their PCD signatures for different ultrasound frequencies and microbubble types in order to ensure safe BBB opening. Here we propose to extend and upgrade the use of PCD in order to monitor real-time the BBB opening treatment in non-human primates.

There are two critical requirements for this technique before undergoing clinical applications. 1) The procedure needs to be proven accurate in order to deliver therapeutic agents only in the desired region of the brain. 2) It also needs to be proven safe. This encompasses determining a reliable set of acoustic parameters, monitoring the treatment in real time and understanding the BBB closing timeline. These questions have been addressed in part for an MRI-based phased array of ultrasound transducers that was developed for treatment of brain tumors [15]. Here we address these questions for our easy-to-use 500-kHz single-element transducer setup. This is particularly important because the system operates outside of an MR scanner. Hence, online monitoring of safety and opening volume in combination with reliable targeting will further increase independence from MRI-based targeting and verification of the opening. This manuscript is meant to pave the way for a long-term study of chronic use of focused ultrasound BBB opening. The main focus of the work presented here is about efficacy and targeting and safety studies are currently ongoing in both mice [16] and monkeys [17].

## Methods

### Ethics statement

All methods were approved by the Institutional Animal care and Use Committee at Columbia University and the New York State Psychiatric Institute. Monkeys had daily interactions with humans as well as two subjects being paired with each other. All monkeys were housed in a room with 18 other monkeys on a 12 hour light dark cycle. Monkeys were fed a diet of vitamin enriched dry primate biscuits daily and were either provided with 1L of water or worked for their water via behavioral tasks until they were satiated. A fruit treat was given to them at the completion of each day along with various enrichment items including a larger play cage with a swing. Before being placed in the stereotax for sonications monkeys were sedated with ketamine for placement of the intubation tube and IV line and then isofluorane was used to keep them unconscious during the procedure. No animals were sacrificed for the purpose of this study.

**Figure 1:**
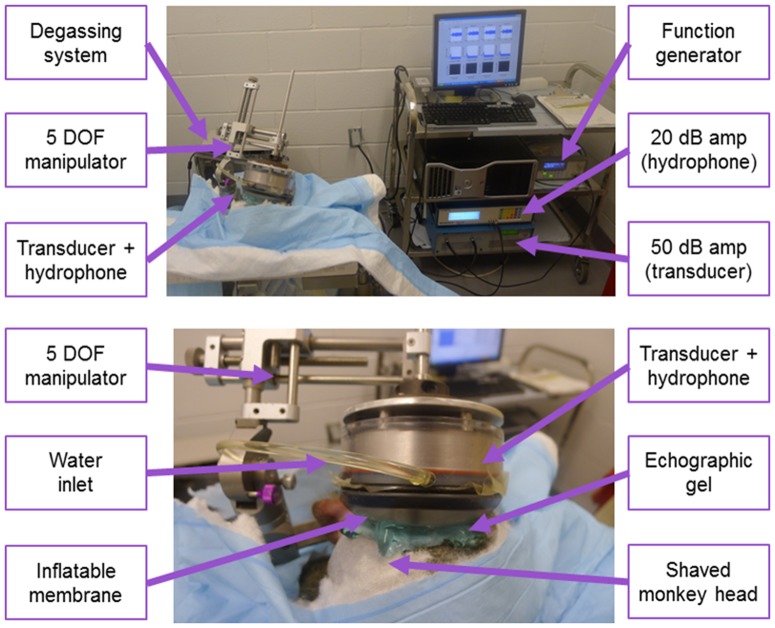
Preclinical setup. Top picture is showing a large view of the operating room. On the right, the PC and amplifiers are used to drive the transducer-hydrophone assembly. The degassing system (vacuum pump+water circulation pump) ensures a constant flow of degassed water for acoustic coupling. The transducer-hydrophone assembly is mounted on a manipulator with 5 degrees of freedom (x, y, and z position of the focus, as well as two approach angles: azimuth and elevation). Bottom picture is depicting a close-up view. The membrane can be inflated regulating the water flow thanks to the degassing system. This ensures a maximal acoustic transmission in the animal brain.

**Figure 2:**
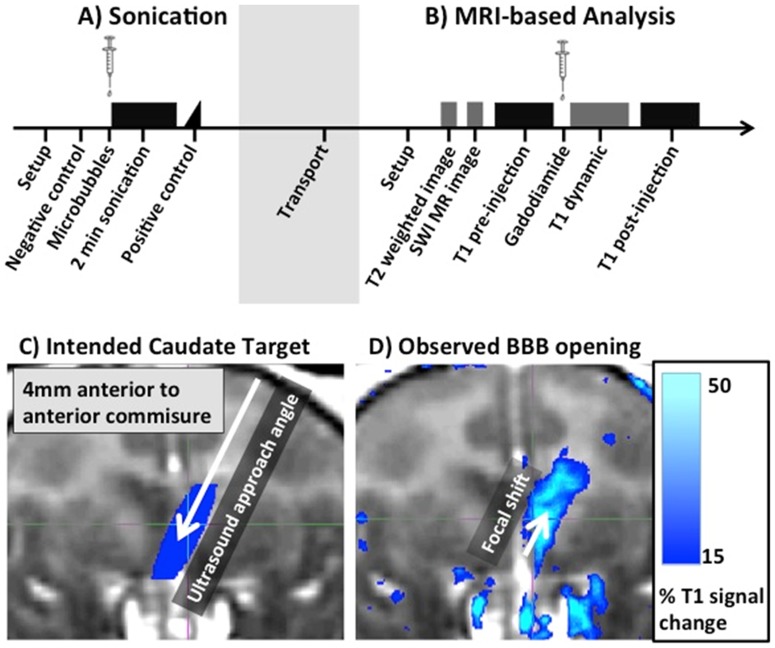
BBB opening procedure overview. (**A&B**) Timeline of sonication experiment with subsequent MRI-based verification. Briefly, the animals are sonicated for two minutes using a 500-kHz focused ultrasound transducer following the systemic injection of microbubbles. The opening location is then analyzed using contrast-enhanced T1 images (see D for details). Additional clinical scans were performed to detect potential damage. (**C**) Geometric ultrasound focus overlaid on a T1 structural scan in stereotaxic coordinate frame. Due to the geometry of the ultrasound transducer, the focal region is elongated along the axis of ultrasound propagation. Here the ultrasound was applied at an angle of 26° from the upper right to provide a close to normal incidence angle of the ultrasound and skull. (**D**) Increased blood-brain barrier (BBB) permeability for the T1 contrast agent gadodiamide following a single sonication of left caudate. Brighter colors indicate regions where gadodiamide was able to diffuse across the BBB into the brain tissue. The remaining regions of increased T1 signal indicate asymmetric vasculature. Note the close alignment between intended (C) and actual location (D) of the BBB opening. The axial shift in location of the BBB opening towards the transducer is close to the value predicted from in-vitro experiments.

The ultrasound procedure has been described in greater detail elsewhere [10], [11]. The series of sonications presented in this study were performed on two male rhesus monkeys (Macaca mulatta) named O and N. Each animal was treated every two weeks. Monkeys were positioned in a stereotaxic frame (Figure 1) under general anesthesia. Sedation was done using ketamine 5–15 mg/kg IM, and anesthesia was done with isoflurane 1–4% inhaled. A 500-kHz ultrasound transducer was attached to a Kopf stereotaxic manipulator to enable targeting of the ultrasound focus in stereotaxic coordinates (see below for details). Once the animal, the targeting coordinates, and the FUS system were set, negative control sonications were performed in the absence of microbubbles (see Figure 2 and below for details). After IV injection of monodispersed 4–5-µm microbubbles that were manufactured in-house and size-isolated using differential centrifugation [18], the animals were sonicated for a total duration of 2 minutes with an estimated focal maximum pressures ranging between 0.20 and 0.30 MPa. Those microbubbles are generally used as ultrasonic contrast agent and they are evacuated very rapidly from the system through the lungs and kidneys (half-life time in humans: 5–10 minutes typically). Post-sonication controls in the presence of microbubbles were performed immediately after the treatment (see below for details). Subsequently, the location of the BBB opening was determined using contrast-enhanced T1 images (see below).

### FUS setup

A 500-kHz center frequency focused ultrasound transducer was used for this experiment (H-107, Sonic Concepts, WA, USA). *In vitro* pressure measurements were realized in another study [12]. This study determined the global attenuation (absorption, reflection and scattering) due to the presence of the skull (around −5.7 dB at 500-kHz). The attenuation in the scalp was assumed to be around −0.9 dB/cm and its thickness was estimated to be equal to 0.5 cm. The attenuation in the monkey brain tissue [19] was assumed to be around −0.5 dB/cm and the thickness of this layer was estimated to be equal to 2 cm. Therefore, the emission amplitude was raised by 7.15 dB (approximately a factor of 2.28) compared to the calibration measurements in water to compensate for the energy loss along the path. A flatband, spherically focused hydrophone (Y-107, Sonic Concepts, WA, USA) was positioned through the center hole of the FUS transducer. The two transducers were aligned so that their focal regions fully overlapped within the confocal volume. The hydrophone, which was connected to a digitizer (Gage Applied Technologies, Inc., Lachine, QC, Canada) through a 20-dB amplification (5800, Olympus NDT, Waltham, MA, USA), was used to monitor real-time acoustic emissions from microbubbles (passive cavitation detection, PCD).

### Targeting

To enable individualized targeting of the ultrasound focus to a particular brain region, T1 weighted stereotaxically aligned structural images were acquired for all animals (same T1 sequence as outlined below). For targeting in stereotaxic coordinate frames we developed an R-based (R Development Core Team 2009) software package (*stereotax.R*) that converted a particular setting of the stereotaxic manipulator (Kopf) into stereotaxic coordinates. The setting of the stereotaxic manipulator is determined by 9 free parameters: the setting of the medio-lateral drive (*ml*), the position of the manipulator on the stereotaxic arm along the anterior-posterior direction (*ap*), the setting of the dorsoventral drive (*dv*), the rotation of the manipulator around the z-axis (*azimuth*), the tilt of the manipulator (*elevation angle*) that could occur either around the ml- or ap-axis (*elevation setting*), the position of the manipulator on the left or right stereotaxic arm (*arm*), the relative alignment of the ml and dv stereotax drives, i.e., was the ml drive positioned anterior or posterior to the dv drive (*stereo*), and finally one degree of freedom that determined the attachment of the ultrasound transducer on the stereotaxic manipulator (*finger*). Based on the setting of the stereotaxic manipulator, the software predicted the focal point and the axis from the focal point to the ultrasound transducer (angle of approach). For visualization purposes, the predicted region of BBB opening around the ultrasound focus was then projected onto the individual stereotaxically aligned T1 image (Figure 2). The software also inverted this procedure: for any desired sonication target (including a desired approach angle) that can be specified in stereotaxic coordinates, the software calculated up to eight different setting of the stereotaxic manipulator that targets this neural structure from the specified approach angle. The optimal approach angle was determined by the user. For the experiments reported here, we set the approach angle to provide a close to perpendicular incidence angle between ultrasound beam and skull.

### Verifying the BBB opening with contrast-enhanced MRI

After sonication, the anesthetized animals were transported to the MR facility where T2 and T2 FLAIR images were taken to detect any potential damage caused by the sonication. The integrity of the BBB was tested using the T1 contrast agent gadodiamide (Omniscan™) that is typically used to visualize the break-down of the BBB in neurological disease [20]–[22]. To that aim, a high-resolution structural T1 image was recorded prior to the injection of gadodimide (T1 Pre; 3D Spoiled Gradient-Echo, TR/TE = 20/1.4 ms; flip angle: 30°; NEX = 2; in-plane resolution: 1×1 mm^2^; slice thickness: 1 mm with no interslice gap). 30 min after injection of 0.15 ml/kg gadodiamide IV, another T1 image was acquired using identical scanning parameters (T1 Post). As gadodiamide does not cross the intact BBB, increased T1 signal strength will be found in vessels or regions with increased BBB permeability [20]–[22]. 3D T2-weighted sequence (TR/TE = 3000/80 ms; flip angle: 90°; NEX = 3; spatial resolution: 400×400 mm^2^; slice thickness: 2 mm with no interslice gap) and 3D Susceptibility-Weighted Image (SWI) sequence were applied (TR/TE = 19/27 ms; flip angle: 15°; NEX = 1; spatial resolution: 400×400 mm^2^; slice thickness: 1 mm with no interslice gap) were used.

### Data Analysis

T1 pre and T1 post images were registered to the individual stereotaxically aligned T1 image using FSL's FLIRT routine [23]. To estimate gadodiamide concentration [Gd]_c_ we divided the post T1 image by the pre T1 image (*post/pre*). The *post/pre* image highlights regions of increased T1 contrast following the injection of gadodiamide. This includes regions of interest where the BBB was opened, but also any vessels or other regions with high blood volume such as the pial surface. The post/pre image was then flipped such that the left hemisphere overlaid the right hemisphere. The un-flipped image was then divided by the flipped image. This procedure removed activations due to high [Gd]c in voxels with high blood-volume, as long as the regions were symmetric between the hemispheres. The resulting image that formed the basis of our analysis highlights increased the [Gd]c in the sonicated region as well as some residual artificial activation mainly due to the asymmetric vasculature.

**Figure 3:**
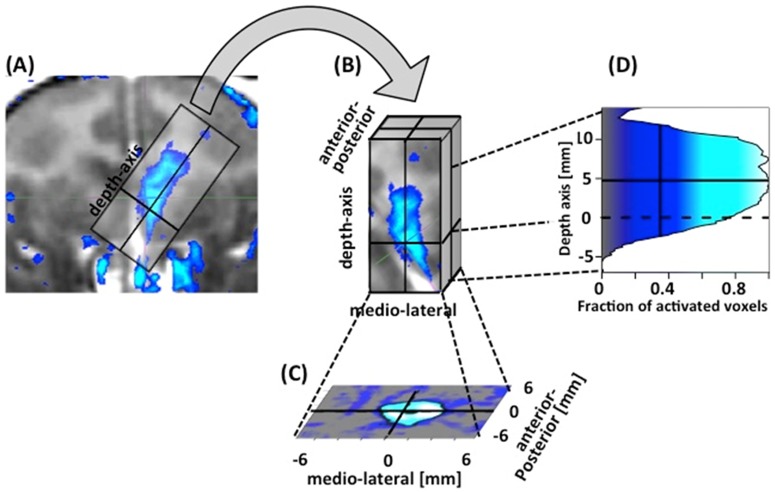
Quantification of targeting accuracy. After calculating the raw result image that provides a normalized estimate of the increase in T1 contrast (**A**), the image is shifted and rotated in to a new coordinate frame (**B**) whose origin is defined by the coordinates of the intended target, and the z-axis corresponds to the approach angle. A voxel is considered opened if its T1 value was enhanced by ≥10%. The in-plane targeting accuracy was assessed by averaging the fraction of opened voxels across the z-axis (**C**). Targeting in the depth axis along the ultrasound beam was quantified by collapsing across the x- and y-axis (**D**).

**Figure 4:**
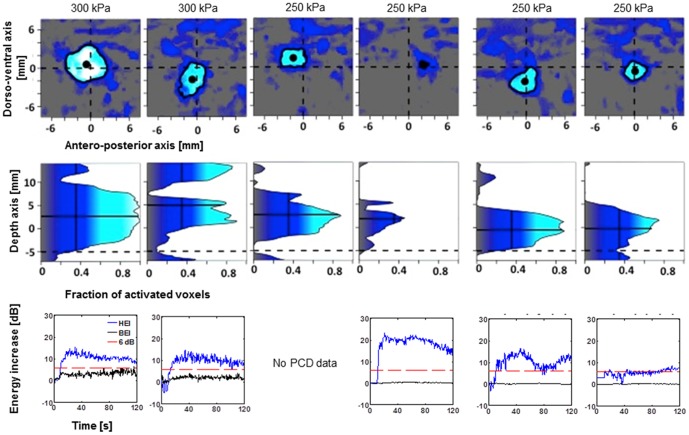
Targeting accuracy for 6 (4+2 for two monkeys O and N) sonications of caudate nucleus. The panels in the first row show the color-coded fraction of activated voxels (>10% enhancement of T1 signal) as a function of medio-lateral and antero-posterior deviation from the intended focal point in the x-y-plane. The panels collapse across voxels that are between −5 and 10 mm in depth from the intended depth. In all instances the opening of the BBB either overlaps with or is in immediate vicinity of the intended target. To quantify targeting accuracy along the direction of the ultrasound propagation, panels in the second row show the fraction of activated voxels collapsed around a 2 by 2 mm square region around the measured focal point (block dots in panels in A). The dotted horizontal line corresponds to the depth of the geometric ultrasound focus. As predicted from in-vitro experiments, the actual focal depth (solid horizontal line) is shifted ∼5 mm towards the ultrasound transducer. Panels in the third row depict the backscattered acoustic energy of the microbubbles excited in the ultrasound focus as a function of time from injection of the microbubbles. The blue line to the desired harmonic oscillations of the microbubbles (HEI) that have been associated with safe BBB opening. The black line corresponds to inertial cavitation (BEI) that has been linked to extravasation of red blood cells and tissue damage. The red line corresponds to the BEI detection threshold.

**Figure 5:**
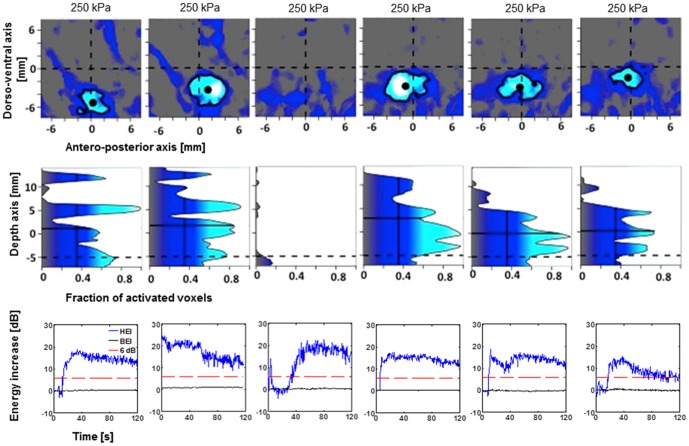
Targeting accuracy and PCD responses for 6 sonications of putamen in animal one. Conventions as in Figure 4. The PCD for sonication 12 06 23 shows immediately elevated HEI values because by accident, the microbubbles were injection before sonication onset.

**Figure 6:**
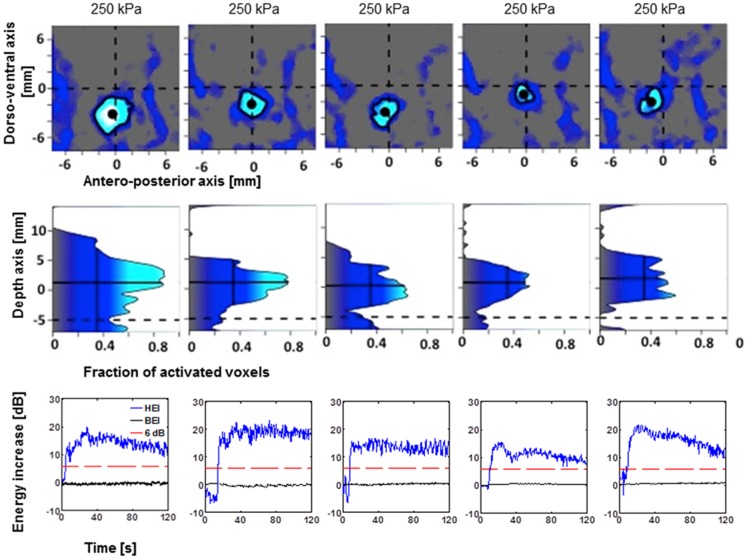
Targeting accuracy and PCD responses for 5 sonications of putamen in the second animal. Conventions as in Figure 4.

To assess the targeting accuracy, the resulting image was rotated and shifted into a new coordinate frame where the origin was defined as the predicted location of the ultrasound focus, and the z-axis corresponded to the approach angle (Figure 3). We selected a region of interest around the origin corresponding to ±7.5 mm in the x- and y-direction, and −5 to +12 mm along the z-axis. A voxel was defined as being “opened” when the T1-enhancement exceeded a threshold of 10%. The total volume of the BBB opening was quantified as the volume of the opened voxels in the region of interest around the sonication target. The fraction of opened voxels was then averaged across the z-axis. Based on this two-dimensional x-y map, the region of the opening was defined as pixels with more than an average of 35% of opened voxels (black contour line). The observed center of the sonication in the x-y-plane was defined as the center of mass of the region of the opening (black dot in Figures 4, 5 and 6). The targeting error in the x-y plane was defined as the difference of the observed position of the opening from the theoretical position of the geometric focus. Similarly, targeting accuracy along the axis of propagation of the ultrasound was assessed by averaging the fraction of opened voxels across the x- and y-axis. The averaging was restricted to voxels within a square region of ±2 mm around the observed xy-center of the sonication. The center of the sonication along the z-axis was defined as the center of gravity of the bins with more than 35% opened voxels. The targeting error along the z-axis was defined as the difference between the observed center of the sonication along the z-axis and the predicted focal depth. The predicted focal depth was assumed to be the geometric focal depth plus 5 mm due to the focal shift induced by the skull.

### Real-time monitoring

The monitoring technique is based on the evolution of the frequency content of the backscattered acoustic signal. Because bubble oscillations along the acoustic excitation are non-linear (stable cavitation) the PCD will detect harmonic modes in the frequency spectrum. Bubble collapse and jet, more generally described as inertial cavitation, induces broadband noise. Therefore, detection of broadband response is the signature of inertial cavitation. A previous study [14] has shown that using 4–5- µm monodispersed microbubbles, inertial cavitation was not required to open the BBB. Also, stable cavitation alone has never been associated with any tissue damage [14]. The frequency spectra of backscattered acoustic emissions were used to infer the cavitation-behavior of the micro-bubbles in the focal region. In order to remove the harmonic (nf, n = 1, 2, …,6), sub-harmonic (f/2) and ultra-harmonic (nf/2, n = 3, 5, 7, 9) frequencies produced by stable cavitation [12], the response within a 300-kHz bandwidth around each harmonic and 100-kHz bandwidth of each sub- and ultra-harmonic frequency were filtered out in order to obtain the broadband signal. This was performed within the 0.6–5.2 MHz frequency band in order to avoid perturbation induced by the fundamental frequency and to take in to account the growing attenuation of the signal along the frequency. From those sets of two spectra, both the broadband and total energies (respectively *ε^broadband^* and *ε^total^*) are computed by summing the spectral amplitudes (s) on the defined frequency range as follows:
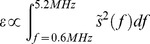



Two metrics are then defined as indications of inertial or stable cavitation by analyzing the differences between backscattered with and without bubbles. The broadband energy increase (BEI) from the negative control level (without microbubbles) was monitored as an indication of inertial cavitation and was defined as follows:
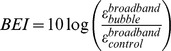



The harmonic energy is obtained by subtracting the broadband energy to the total energy. The harmonic energy increase (HEI) is an indication of stable cavitation and was defined as follows:
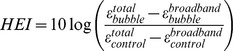



The energy increase of the control signals were defined as the average value of the 2 second long negative control sonication that was taken right before injecting the bubbles but otherwise used the exact same ultrasound parameters as the treatment sonication.

Immediately after the treatment sonication, a series of 2-sec positive control sonications were performed while microbubbles were still in circulation. The positive controls used pressures between 0.05 and 0.35 MPa. Except for the shorter duration and variable pressures, they used the exact same sonication settings that were applied for the treatment sonication. The aim of the positive controls was to describe the relationship between ultrasound pressure and the harmonic/broadband energy increase. There were 8 testing sets done in the study. We calculated the mean HEI over the entire sonication to relate stable cavitation to the observed size of the BBB opening.

## Results

This study reports the results of a series of 17 sonications targeting the caudate nucleus (6) and the putamen (11) in the left hemispheres of two macaque monkeys. The analyses are focused on targeting accuracy, the relationship between PCD response and BBB opening volume as well as safety of the procedure. In addition, one exploratory study examined the duration for which the BBB remains open after the sonication.

### Targeting Accuracy

Figure 2 shows a typical result of BBB disruption using T1-weighted MR imaging and gadodiamide MR contrast agent. The image on the left depicts the theoretical position of the ultrasound focus. The image on the right renders regions where the T1 contrast agent gadodiamide was able to diffuse to the brain parenchyma as a result of BBB opening (see Methods for details). This figure highlights the good qualitative agreement between the intended target of the ultrasound focus and the actual region of increased BBB permeability.

In order to quantify the targeting accuracy of the method, the processing described in Figure 3 was performed for each experiment (see Methods). The individual plots for lateral and axial targeting accuracy are depicted in Figure 4 for caudate targets of both animals. Figures 5 and 6 provide identical plots for the putamen sonications in the two animals, respectively. These results prove the reproducibility and targeting precision of the FUS technique. First, we quantified the *targeting accuracy* by averaging the relative focal position for all sonications and animals. The mean focal point was 0.2±1.0 mm posterior to the intended target (all results are reported as mean±standard deviation in mm). This difference did not reach significance (t-test, p>0.05). The observed focal point was significantly ventral to the intended target (1.9±1.7 mm; t-test p<0.05). Further, the mean focal point was significantly shifted towards the ultrasound transducer (1.4±1.4 mm, t-test, p<0.05). It is important to note that predicted focal depth was defined as the depth of the geometric ultrasound focus plus 5 mm (*i.e.*, shifted towards the ultrasound transducer). The 5 mm shift was added to account for the shift of focal depth that was measured *in vitro* with immersed skull plates [12]. Hence, our results demonstrate the close correspondence between the *in vitro* and *in vivo* measurements. However, they imply a somewhat stronger focal shift was observed in our *in vivo* experiments.

The reliability of the sonication procedure was assessed as the *mean targeting error* (absolute distance from intended target). The mean targeting error over all sonications in the lateral plane was 2.5±1.2 mm. Mean targeting error in the axial direction was 1.5±1.3 mm. Combined lateral and axial error averaged 3.1±1.3 mm.

We further tried to dissociate random errors due to day-to-day fluctuations from systematic targeting errors that could be specific to a particular animal and/or target. To quantify the systematic targeting error we averaged the location of the focal point for both targets and both animals separately. The mean systematic lateral targeting error was 1.8 mm. Mean systematic axial targeting error was 1.4 mm. Combining the lateral and axial error we observed a mean systematic targeting error of 2.7 mm across all four targets (2 targets in 2 animals). We used an analysis of variance to test whether targeting accuracy differs as a function of the four different groups of sonications (two targets in two animals). Neither anterior-posterior nor axial position (relative to the intended target) differed as a function of the sonication group. However, we found that dorso-ventral position depended on sonication group (ANOVA, p<0.05). This effect is most likely due to the difference between the two caudate and the two putamen targets. In both animals, the sonications to putamen exhibited a systematic targeting error in the along the dorso-ventral axis. No such systematic targeting error was found in the caudate sonications.

We further quantified the random error, i.e., the absolute distance of the observed focus from the mean focal point over all repetitions with the same target in the same animal. The mean random lateral error was 1.2±0.6 mm. The mean random axial error was 0.6±0.6 mm. Combining lateral and axial error we find a mean random error of 1.5±0.7 mm.

We then quantified the size of the region in which the permeability of the BBB was increased. Averaged over all sonications, the volume of the BBB opening was estimated at 115±44 mm^3^. Larger openings were observed at higher sonication pressures (0.30 MPa, Figure 8A). Moderate openings were observed at lower pressures (0.20 or 0.25 MPa). One sonication at 0.25 MPa failed to elicit any opening (Figure 5). Another sonication at 0.20 MPa only elicited a minimal opening (Figure 4).

### Real-time PCD monitoring

**Figure 7:**
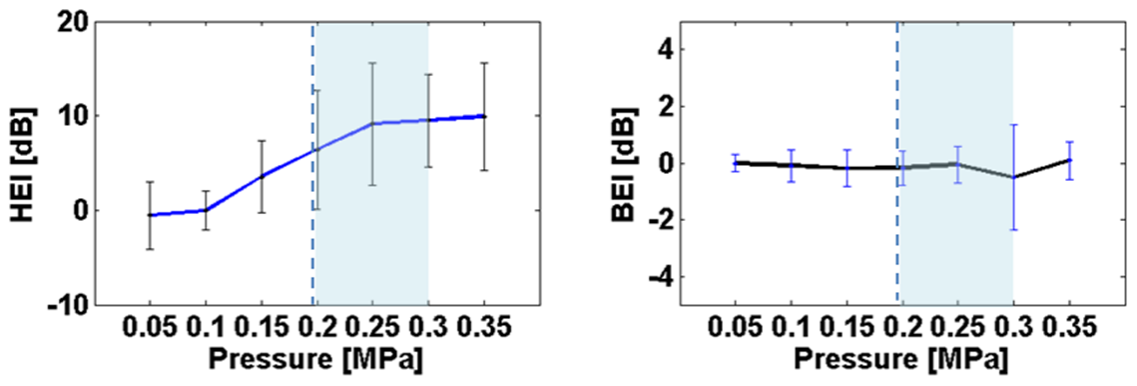
Harmonic (HEI) and broadband (BEI) energy increase plotted as a function of ultrasound pressure. Data was acquired using a series of brief pulses of ultrasound after the main sonication while micro-bubbles were still circulating. The blue dash line corresponds to the lowest pressure at which BBB opening was achieved, and the. The light blue area highlights the pressure range used in this study. The red line corresponds to the ultrasound pressure that would cause BEI to rise above levels that were found to be safe in the current set of sonications.

HEI and BEI monitoring were performed for each experiment in real time. The lower rows in Figures 5–7 render the recorded real-time monitoring for the corresponding sonications. In all but one of the sonications, HEI increased by at least 15 dB during the sonication. This is indicative of stable cavitation of the bubbles in the focal region. The lack of an increase in broadband energy indicates the absence of potentially harmful inertial cavitation. A 6 dB threshold, corresponding approximately to two times average of the negative controls, had been set as a limit of potential damage and was never surpassed.

To characterize the dynamic range of the HEI and BEI responses, we measured acoustic emissions as a function of ultrasound pressure using a series of brief ultrasound pulses of a wide range of pressures (see Methods). Figure 7 shows HEI and BEI as a function of ultrasound pressure. As expected, the HEI starts increasing for lower pressures (0.15 MPa). In contrast, the BEI remains unchanged at 0 dB for pressures up to 0.35 MPa. The HEI seems to reach an asymptote of approximately 10 dB for pressures at and above 0.25 MPa. It is lower than what is shown in the real-time PCD monitoring since this PCD testing was done after the treatment sonication and part of the circulating bubbles were degraded. This analysis defines a window between 0.15 and 0.35 MPa that leads to a reliable increase of harmonic energy while avoiding potentially harmful broad-band energy increase. In this study, pressures were well within this window and ranged between 0.20 and 0.30 MPa.

**Figure 8:**
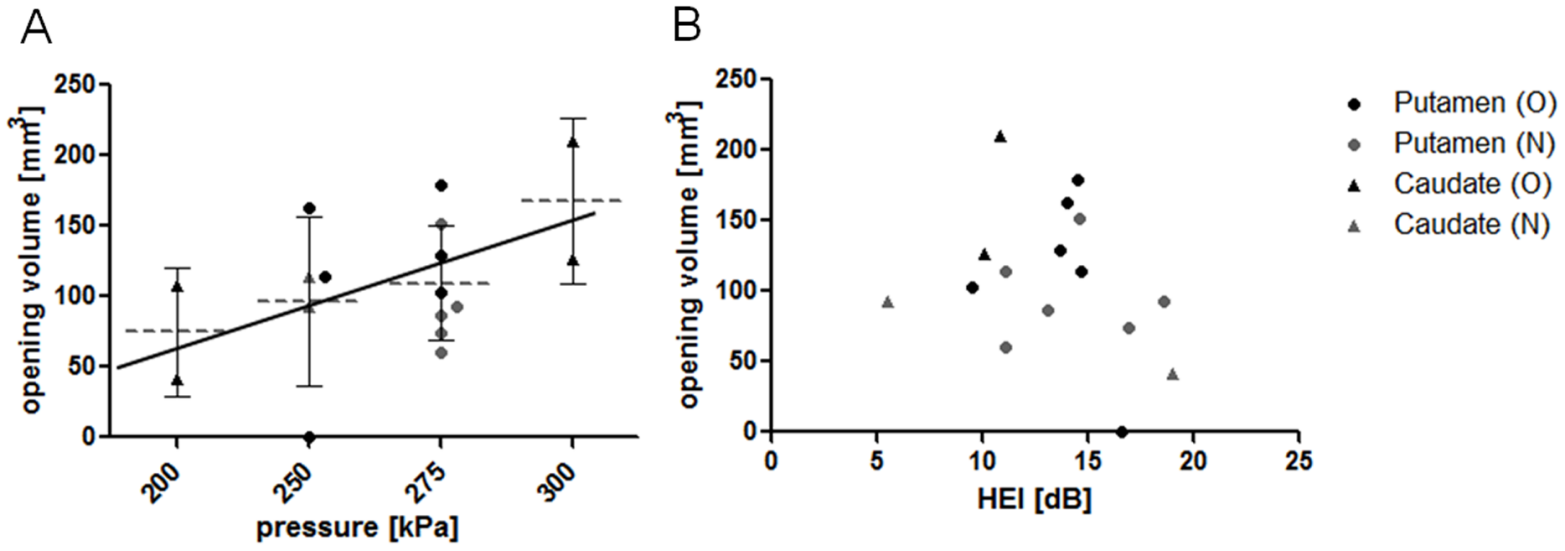
BBB opening volume as a function of pressure (A) and the average harmonic energy increase, HEI (B). Two targets in the putamen and the caudate for two animal subjects (O and N) were marked separately. (**A**) There is a clear relationship between ultrasound pressure and opening size (r = 0.41). Due to the narrow range of pressures and low number of sonications, this effect does not reach significance. (**B**) There is no apparent relationship between average HEI and opening volume.

We then tested whether the online PCD monitoring can be used to predict the success of the sonication and the size of the ensuing BBB opening. To that aim we plotted size of the BBB opening as a function of the mean HEI during the 2-minute sonication period (Figure 8B). Our results show that stronger HEI responses are not indicative of larger BBB opening volume. However, in all but two cases, the presence of HEI went along with a successful BBB opening.

### Safety

**Figure 9:**
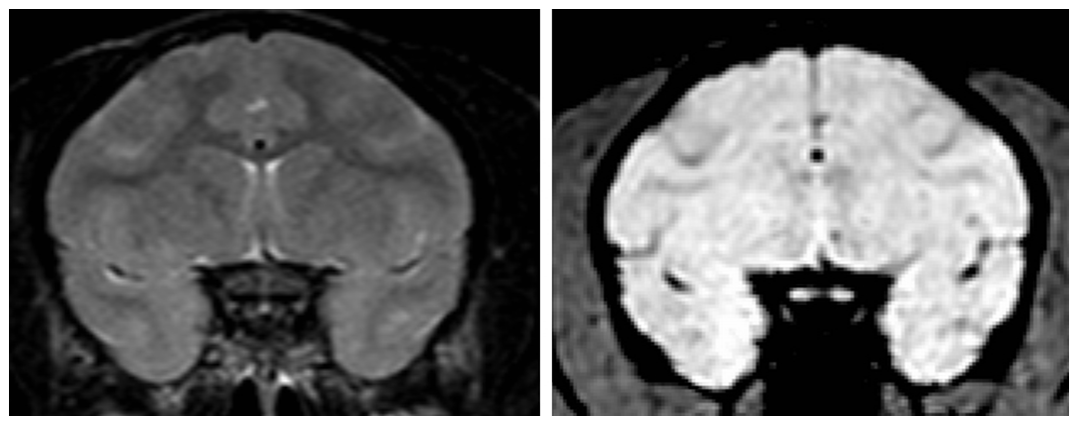
Example of T2-weighted (left) and SWI (right) MR images corresponding to the experiment from Figure 2. Edemas should appear brighter in T2-weighted images; hemorrhages, as well as large vessels should appear in black in SWI images. No damage was detected on any of the experiments performed.

Additional MR imaging sequences (T2-weigthed and SWI, see Methods) were used to assess potential brain damage after the ultrasound procedure. In line with the observed stable cavitation that is indicative of safe in situ ultrasound pressures, neither T2 nor SWI images detected any damage such as edema or hemorrhage in all experiments reported in this paper. Figure 9 shows coronal slices of T2-weighted and SWI images corresponding to the T1-weigthed coronal slices rendered in Figure 2. Qualitatively, we did not notice any post-procedure clinical deficits in activity level, movement or feeding/appetite. As no animal was sacrificed, there was no histological assessment of tissue damage.

### Closing timeline

**Figure 10:**
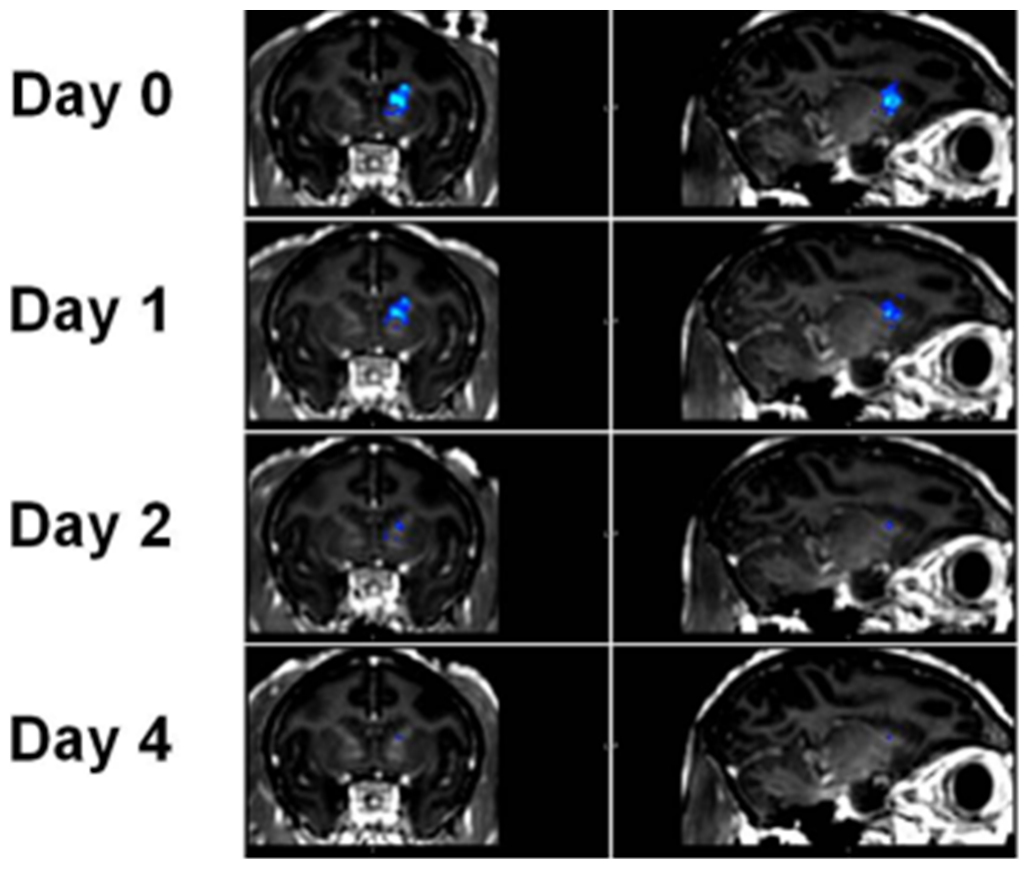
Coronal (left row) and sagittal (right row) T1-weighted MR slices showing the evolution of the BBB opening volume along time. The area with contrast agent diffusion is overlaid in blue. The BBB is restored between day 2 and 4.

**Figure 11:**
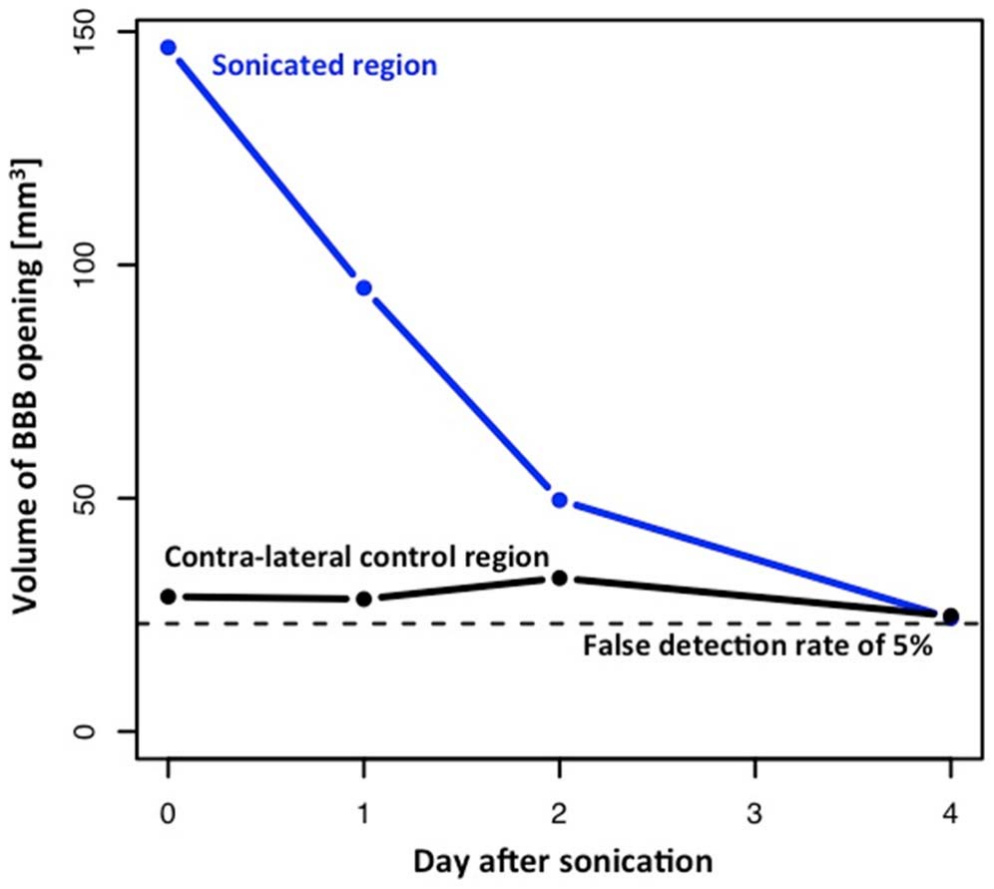
Timeline of BBB closing for a single low-pressure sonication depicted in Figure 10. Voxels with a normalized pre-post enhancement of more than 10% were classified as “opened”. The total volume of opened voxels decreases as a function of time from the sonication. The opened volume in the contra-lateral control region is constant and close to the one predicted by a false detection rate of 5%.

A preliminary experiment to investigate the closing timeline was also performed. As a first step we measured the time course of the BBB closing for a single sonication in one of the macaque subjects. Gadodiamide IV injections along with pre- and post-T1-weighted MR sequences were repeated 1, 2 and 4 days after the initial ultrasound treatment. Coronal and sagittal slices of these experiments can be seen in Figure 10. Standard T1 contrast enhanced imaging and subsequent analyses indicate a clearly visible, average-sized (126 mm^3^) BBB opening. Figure 11 shows the opening volume decreased with time. The BBB was almost completely restored two days after sonication. Experiments in mice have shown that the duration of the BBB opening depends on acoustic and microbubble parameters [24].

## Discussion

The experiments presented in this paper were aimed at testing whether a single spherical transducer at an intermediate frequency of 500-kHz can be used for accurate, repeatable and localized blood-brain barrier disruption in deep subcortical structures. The observed targeting error was sufficiently small (2.5±1.2 mm laterally, 1.5±1.3 mm along depth-axis, 3.1±1.3 mm total) to enable the specific targeting of substructures of the basal ganglia such as the associative or oculomotor caudate. These findings are consistent with and extend our previous *in vitro* and preliminary *in vivo* findings [10], [12]. This technique might be suitable for precise drug delivery applications into different brain structures. The average volume of the BBB opening was estimated at 115±44 mm^3^. As a comparison, the putamen has an approximate volume of 494.8 mm^3^ and the caudate of 451.4 mm^3^ [25].

### Sources of targeting errors

To further reduce the targeting error it is important to analyze potential sources of the error. Here we will briefly discuss three potential factors: errors due to deviation of the geometric focus from the intended target (geometric errors), errors due to the analysis of the focal position (analysis errors), and errors due to deviation of actual ultrasound focus from the geometric focus (ultrasound aberration errors). We will argue that most of the overall error is geometric or analysis errors.

#### Geometric errors

Over the course of the experiments, we repeatedly calibrated the stereotaxic manipulator and the targeting routine. For these calibrations we used a metal rod that was attached to the stereotaxic manipulator in the same way as the ultrasound transducer. The length of the rod was chosen to match the focal length of the transducer and hence its tip corresponded to the location of geometric ultrasound focus (assuming there were no ultrasound aberrations). This setup enabled us to target various know positions, such as the interaural point of the stereotax. These measurements routinely found deviations from the intended target on the order of 1–2 mm. Geometric error arises when the setting on the stereotaxic manipulator that determines geometric focus is off. The position of the geometric focus is determined by the 9 degrees of freedom of the stereotaxic manipulator. Some of these settings are continuous and prone to error. The ml, ap, and dv settings have 1 mm scales in combination with a vernier scale to enable accuracy on the order of a tenth of a millimeter. The azimuth and elevation scales, however, are divided in steps of 5 and 2 degrees, respectively, without an additional vernier scale. This enables accuracy on the order of 1 to 2 degrees. Even small angular deviations may have a big effect on the final position of the geometric focus.

The elevation setting is critical an additional reason: If the approach angle deviates from vertical, gravitational forces perpendicular to the approach angle will grow stronger. These gravitational forces may introduce systematic errors for angled approach vectors. It is interesting to note that the mislocalization in the dorso-ventral direction was strongest for the putamen target, and that this target required a more angled approach. Note that the ventral mislocalization decreases over time (Figure 7 and 8). This may reflect the fact that over the time-course of the experiments reported here we learned to use more force to fasten the set-screws that are responsible to maintain the elevation angle against gravitational pull.

#### Analysis errors

Further, it is important to point out that analysis pipeline used to infer the observed focal point may induce additional small errors. The analysis depends on alignment of pre- and post contrast-enhanced T1 images to a stereotaxically aligned reference image. Small errors may arise during the registration process of the pre- and post images to the reference. Similarly the alignment of the reference image may not perfectly match the intended stereotaxic alignment. In addition, the actual position of the animal in the stereotax may vary slightly on a day-by-day basis. Together these factors could contribute up to 1 mm of the random and/or systematic targeting error. Further, the fractional enhancement of the post- relative to the pre- image is based on noisy T1 MRI images. While it is difficult to quantify, this factor will certainly contribute to the overall targeting error.

#### Ultrasound aberration errors

The above data and analyses argue that most of the error may easily be explained by geometric and analysis factors. However, mislocalization in the axial direction is known to occur due to ultrasound aberrations based on in-vitro measurements with immersed skull plates [12]. Our findings closely replicate the in vitro findings. On average we observed a 6.5 mm focal shift, compared to the predicted 5 mm focal shift. At this point it is not clear if the additional 1.5 mm are due to different ultrasound aberrations *in vivo* or if they are due to geometric and analysis error.

### Real-time PCD monitoring

Real-time monitoring based on the frequency content of the backscattered signal was performed to classify the cavitation behavior and hence establish the success and safety of the sonication. Measuring the cavitation spectrum is helpful to verify that the microbubbles are correctly excited *in situ*, *i.e.*, non-linear resonance along the ultrasonic frequency without broadband noise signature of bubbles collapsing or micro-jet streaming (inertial cavitation). This translates into a significant HEI (between 15 dB and 25 dB) and no BEI. During all experiments performed in this paper (pressures at or below 0.3 MPa) only stable cavitation was observed. Therefore, the PCD monitoring indicated that the procedure would be safe and successful. Also, the HEI has been found to be indicator of the success of the BBB opening in these initial findings. For the cases with an average HEI higher than 5 dB, there was 94% (15/16) of success. The correlation between the HEI and the opening volume in Figure 8B was not high since we focused on a small range of pressures (0.20–0.30 MPa). It might be a more reliable predictive metric if the correction of the skull attenuation could be made.

### Future directions

#### MRI-independent targeted BBB opening

Focused ultrasound can be used to temporarily disrupt the integrity of the blood brain-barrier in specifically targeted brain regions of rodents and monkeys. In the near future, focused ultrasound may allow clinicians to deliver drugs to specific neural targets. However, current clinical ultrasound setups comprise multi-phased ultrasound transducer arrays located inside an MR scanner. This restricts the use of ultrasound to highly specialized clinical settings. Here we use a low-tech single-element 500-kHz spherical transducer ultrasound setup that has the potential to overcome this limitation. The system was specifically designed in principle to be portable, and uses a stereotaxic targeting procedure to make it independent of MR guided targeting. The current paper addressed two essential questions to verify that the system can indeed be used independent of an MR scanner. First, we have shown that the stereotaxic targeting procedure is accurate and reliable. Second, we tested whether the success of the sonication can reliably be inferred using real-time passive cavitation spectral analysis. While successful sonications were almost always accompanied by a 10–15 dB HEI, we found no correlation between HEI and opening volume. Additional experiments are needed to establish a closer link between the PCD monitoring and the outcome of the sonication.

In summary, these findings show that our portable system can safely and reliably be used to open the BBB in specific brain regions of the macaque monkey, largely independent of MRI-guided targeting and/or verification. Hence, in principle, this system has the potential to provide non-invasive targeted brain-drug delivery to a broad patient base in less specialized clinical settings (e.g., outpatient clinics; community hospitals). It is of course important to note that targeting accuracy can be increased by using an individual stereotaxically aligned T1 image. This, however, constitutes a one-time procedure and all following sonications would be completely independent of MRI.

#### Time-course of BBB closing

The ultimate goal of the ultrasound method is to deliver pharmacological agents to specifically targeted neural substrates. The results and analyses outlined above show that the single-element FUS method can be used to accurately and reliably target sub-structures of the basal ganglia. However, there still remain a number of questions that need to be answered before the method can be used to deliver drugs to those neural targets in human subjects. In particular, it is still not known how long the BBB will stay open before it regenerates and prevents the passage of molecules from the blood to the brain. This is important for two reasons: First, this determines the window of opportunity during which drugs can be delivered. On the other hand, this determines how long the brain region in question will be exposed to other substances that usually would not cross the intact BBB. Previous studies in mice have indicated that the duration of the BBB opening depends on the precise sonication parameters such as ultrasound pressure and microbubble size. The duration of the BBB opening can range between 12 hours and 5 days [24]. However, to date it is not known whether the same relationship between ultrasound parameters and BBB opening duration also holds for human subjects. Due to the closer similarity between brain structures of the two species, these measurements may provide a more accurate idea about what time course to expect in the human brain. The results from a single exploratory analysis indicated that an average-sized BBB opening (∼126 mm^3^) with moderate in situ ultrasound pressures (0.30 MPa) and 4–5-µm monodisperse microbubbles takes between 2 and 4 days to close. This finding is in agreement with previous work performed in mice [24]. However, a more thorough set of experiments is needed to verify the inter-species agreement over a wider set of parameters.

### Safety of repeated sonications and drug delivery

As stated in the introduction, ongoing chronic studies are being performed on both mice and monkeys. These studies will include safety investigation of repeated openings over 6 months in mice [16] and complete behavioral testing in monkeys [17]. Future studies will also been needed to determine whether therapeutically relevant levels of the drugs can be maintained after crossing the BBB.

## Conclusion

The findings of this study demonstrated that a single spherical transducer operating at an intermediate frequency of 500-kHz can be used for accurate, repeatable and localized blood-brain barrier disruption in deep subcortical structures. This constitutes an important first step towards developing a non-invasive targeted drug-delivery system. This method shows unique potential for clinical treatments of neurodegenerative diseases and also for neuroscientists, who aim at dissecting the function of deep regions of the brain such as the basal ganglia. Real-time monitoring was developed and may be a trustworthy safety measure during BBB opening. Preliminary results show that using our typical set of acoustic parameters, the integrity of the BBB was restored after approximately two days.
